# How to Synchronize Longitudinal Patient Data With the Underlying Disease Progression: A Pilot Study Using the Biomarker CRP for Timing COVID-19

**DOI:** 10.3389/fmed.2021.607594

**Published:** 2021-07-08

**Authors:** Martina A. Maibach, Ahmed Allam, Matthias P. Hilty, Nicolas A. Perez Gonzalez, Philipp K. Buehler, Pedro D. Wendel Garcia, Silvio D. Brugger, Christoph C. Ganter, The CoViD-19 ICU-Research Group Zurich, The RISC-19-ICU Investigators, Michael Krauthammer, Reto A. Schuepbach, Jan Bartussek

**Affiliations:** ^1^Institute for Intensive Care Medicine, University and University Hospital Zurich, Zurich, Switzerland; ^2^Department of Quantitative Biomedicine, University and University Hospital Zurich, Zurich, Switzerland; ^3^Department of Infectious Diseases and Hospital Epidemiology, University and University Hospital Zurich, Zurich, Switzerland

**Keywords:** COVID-19, longitudinal data, synchronization, subgroup comparison, risk stratification, biomarker, digitalization, patient trajectories

## Abstract

The continued digitalization of medicine has led to an increased availability of longitudinal patient data that allows the investigation of novel and known diseases in unprecedented detail. However, to accurately describe any underlying pathophysiology and allow inter-patient comparisons, individual patient trajectories have to be synchronized based on temporal markers. In this pilot study, we use longitudinal data from critically ill ICU COVID-19 patients to compare the commonly used alignment markers “onset of symptoms,” “hospital admission,” and “ICU admission” with a novel objective method based on the peak value of the inflammatory marker C-reactive protein (CRP). By applying our CRP-based method to align the progression of neutrophils and lymphocytes, we were able to define a pathophysiological window that improved mortality risk stratification in our COVID-19 patient cohort. Our data highlights that proper synchronization of longitudinal patient data is crucial for accurate interpatient comparisons and the definition of relevant subgroups. The use of objective temporal disease markers will facilitate both translational research efforts and multicenter trials.

## Introduction

The rapid spread of the corona virus disease 19 (COVID-19), caused by the SARS-Cov-2 virus, imposes a heavy burden on public health systems around the world. A substantial number of patients show a severe disease progression possibly caused by endotheliitis, gas diffusion impairment and organ ischemia (1, 2). Current research efforts focus on the identification of predictive indicators that allow closer supervision and targeted intervention in high-risk patients. As a hyper-activated immune response might act as a driving factor for severe COVID-19 progression (1), ratios between neutrophils and lymphocytes (NLR) (3), lymphocyte counts alone (4) and elevation of specific cytokines among other laboratory values (3, 5–7) have been proposed as markers for initial patient risk assessment and stratification. Most studies solely compare measurements taken at hospital or intensive care unit (ICU) admission, neglecting the enormous potential of continuous longitudinal data obtained throughout hospitalization (3–8). This is especially detrimental for the most severe patients, as this high mortality risk group could benefit the most from a more detailed separation into different disease progression subgroups. However, the pooling of longitudinal data requires a temporal marker to align individual patient trajectories.

**Figure 1:**
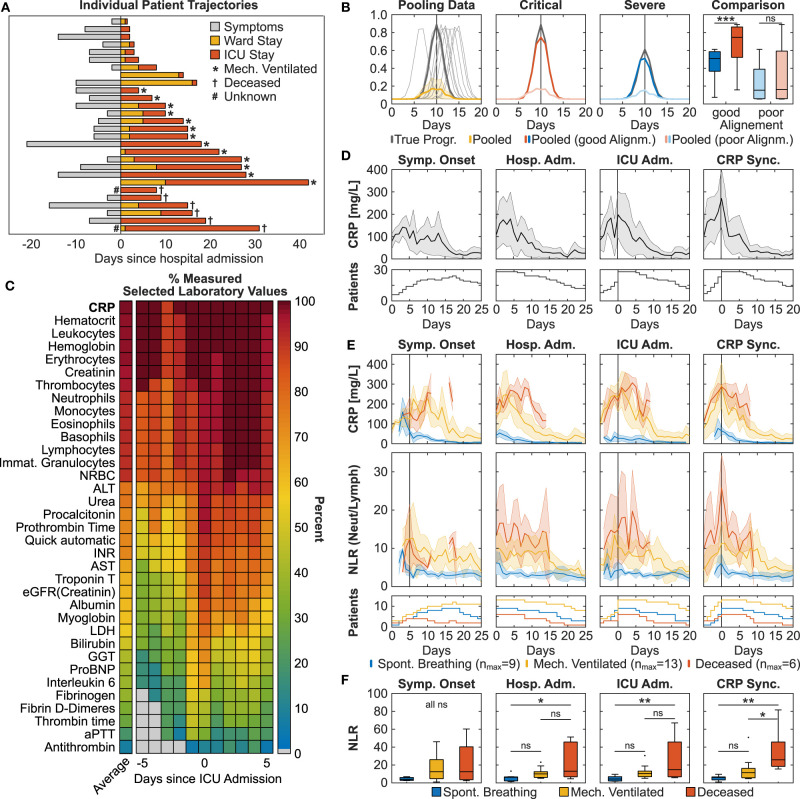
Pathophysiological synchronization of COVID-19 trajectories improves subgroup distinction. **(A)** Individual patient trajectories of 28 severe ICU COVID-19 patients. **(B)** Simulation illustrating the effect of pooling temporally shifted data. Left: Simulation of peaking biomarker progression, 100 identical time courses with maximum value scattered around day 10 (normally distributed, σ = 1.5, one sampling per day). Dark gray line: true progression without temporal scatter; light gray lines: 10 randomly selected curves of the simulation; yellow line: median ± MAD (median absolute deviation) of the 100 simulated curves. Middle panels: Gray lines represent two identical curves that differ in height by 50%. Light colored curves: σ = 1.5, dark colored curves σ = 0.75. Right: Boxplot comparison of the simulated curves in the middle panels at time point 10 days. **(C)** Heat plot of average measurement frequency around ICU admission. **(D,E)** Time course of CRP overall **(D)** and CRP and NLR in severity subgroups **(E)**. Synchronization based on onset of symptoms resulted in the exclusion of two deceased patients due to unclear data. Data is shown as median ± MAD. Curves are cut-off when data of fewer than three patients was available. The respective patient numbers are shown in the bottom panels. **(F)** Subgroup comparison of each alignment method at time point 0 for CRP_max_-based, hospital and ICU admission-based and time point +5 days for onset of symptoms based alignment (indicated by the gray line in subfigure **E**). Multiple comparison testing with Tukey *post-hoc* test was performed on single time points. ns = not significant, *p ≤ 0.05, **p ≤ 0.01, ***p ≤ 0.001.

During the first wave of the corona virus pandemic, patient comparisons were often made based on clinical time points such as “onset of symptoms,” ”hospital admission,” or “ICU admission” (3–8). Group comparisons based on these clinical markers might however result in the description of false differences, for example by comparing patients in late disease stages with patients in early disease stages (8), or blurring of actual differences due to temporal misalignment (Figures 1A,B). An ideal disease timer should be an objective biomarker that can provide an early indication of disease progression and should be measured routinely in most hospital settings. In this pilot study, we compare different alignment methods and show that C-reactive protein (CRP) can be used to synchronize individual patient trajectories to the underlying pathophysiology of COVID-19.

## Methods

### Inclusion Criteria, Ethics Approval, and Consent to Participate

We included all COVID-19 patients admitted to the University Hospital Zurich between March and July 2020 with age older than 18 years that required ICU treatment. We excluded patients with objection to the further use of medical data in research, and patients that where transferred from other hospitals. The data used in this manuscript was routinely collected during hospitalization. Whenever possible, we obtained a written informed consent of the patients (or relatives) for the further use of their medical data for research. This study has been approved by the cantonal ethics committee of Zurich.

### Time Series Analysis

We used MATLAB (The MathWorks, Inc. USA) to analyze and visualize longitudinal patient data. The MATLAB function findpeaks.m was used to identify the first local CRP maximum (CRP_max_). We developed custom scripts for synchronization of the time series with the temporal markers.

### Predictive Modeling

Two feature vectors were generated for each patient containing the mean values of CRP, relative neutrophils and lymphocytes of a time window anchored on either ICU admission or on CRP_max_. We followed a stratified 5-fold cross-validation scheme, where each fold was defined as a distinct 80–20% train-test split. Within each fold, hyper-parameter selection was performed in the training set with a stratified 4-fold cross validation. For each fold multiple logistic regression models were trained using varying hyper-parameters such as regularization type (*l*_1_, *l*_2_) (9), regularization value in the interval of (10^−4^ 10^4^), optimizer [LBFGS (10), SAGA (11)] and with or without class weighting. The best models as determined by F1-macro score on the 4-fold cross validation were then tested on the test split. Since our retrospective patient classification was done at ICU discharge, only values before outcome classification were considered for the construction of the feature vectors. This lead to the exclusion of 2 patients from the spontaneously breathing subgroup.

### Statistical Analysis

Statistical testing was performed with R version 3.6.3. Multiple comparison testing with a Tukey *post-hoc* test was performed on single time points comparisons. Patient characterization data was tested by ANOVA for normally distributed data, Kruskal-Wallis test for non-normally distributed data and χ^2^-test for binary data. A mixed linear regression model analysis was performed for CRP_max_ and ICU shifted data, with likelihood ratio test for overall and Satterthwaite approximation for subgroup analysis.

## Results

A comparison of individual patient trajectories in our cohort of 28 critically ill COVID-19 patients admitted to the ICU of the University Hospital Zurich (Table 1) revealed considerable interpatient variability (Figure 1A): Out of the 28 patients, 8 were directly transferred to the ICU upon hospital admission and 5 additional patients were transferred to the ICU only one day after hospital admission. Based on this data alone, it is evident that interpatient comparison at hospital or ICU admission was biased in our ICU COVID-19 patient cohort. Likewise, onset of symptoms showed a high variation (7.65 ± 8.49 days) and was occasionally missing.

**Table 1:** Patient characteristics at ICU admission.

	**Overall (*n* = 28)**	**Spont. Breathing (*n* = 9)**	**Mech. Vent (*n* = 13)**	**Deceased (*n* = 6)**	***p***
**Age**, mean (SD)	65.5 (11.4)	67.8 (12.8)	62.6 (9.5)	68.5 (13.3)	0.46
**Male sex**, *n* (%)	0.8 (0.4)	0.7 (0.5)	0.8 (0.4)	1.0 (0.0)	0.26
**BMI**, mean (SD)	28.1 (4.2)	30.3 (5.5)	27.4 (3.4)	26.4 (2.0)	0.15
**Coronary artery disease**, *n* (%)	17 (60.7)	4 (44.4)	8 (61.5)	5 (83.3)	0.32
**Chronic heart failure**, *n* (%)	8 (28.6)	2 (22.2)	3 (23.1)	3 (50.0)	0.42
**Peripheral artery disease**, *n* (%)	1 (3.6)	1 (11.1)	0 (0.0)	0 (0.0)	0.33
**Arterial hypertension**, *n* (%)	9 (32.1)	1 (11.1)	5 (38.5)	3 (50.0)	0.23
**Diabetes mellitus**, *n* (%)	5 (17.9)	2 (22.2)	2 (15.4)	1 (16.7)	0.92
**Insulin dep. diabetes mellitus**, *n* (%)	11 (39.3)	3 (33.3)	4 (30.8)	4 (66.7)	0.30
**Symptoms to hosp. time**, mean (SD)	5.6 (5.0)	5.9 (4.8)	5.2 (5.8)	6.2 (4.5)	0.92
**Hosp. to ICU adm. time**, mean (SD)	3.0 (5.0)	3.9 (6.2)	3.2 (5.2)	1.3 (1.8)	0.63
**ICU length of stay**, mean (SD)	12.6 (11.3)	8.4 (17.0)	14.5 (7.9)	13.8 (8.8)	0.53
**Apache II**, mean (SD)	17.5 (8.0)	11.8 (7.0)	18.8 (6.9)	23.3 (6.6)	0.01
**SAPS II**, mean (SD)	57.5 (19.9)	43.2 (18.1)	60.6 (17.5)	72.2 (15.4)	0.01
**SOFA**, mean (SD)	13.0 (4.6)	9.9 (4.7)	14.2 (4.1)	15.0 (3.5)	0.04

*Non-binary data is shown as mean (SD) and binary data as mean (%). p-Values indicate one-way ANOVA for normally distributed data, Kruskal-Wallis-test for non-normally distributed data and χ^2^-test for binary data*.

To find an alternative disease timer, we compared the testing frequency of routine laboratory parameters such as the acute phase inflammatory marker CRP, the inflammatory cytokine interleukin 6 (IL-6), myoglobin and cardiac troponin that have previously been correlated to COVID-19 severity (6). We found that IL-6, myoglobin and cardiac troponin were not measured on a daily basis around ICU admission both in our cohort (38.2, 59.7, 62.7% respectively) and in the international RISC-19-ICU registry cohort of critically ill COVID-19 patients (14.6, 9.6, 30.0% in Switzerland and 15.3, 6.7, 28.2% internationally), thereby making them poor candidates for longitudinal data alignment (Figure 1C). In contrast, CRP was measured routinely around ICU admission both in our cohort (98.2%) and in the RISC-19-ICU registry cohort (86.9% in Switzerland, 74.8% internationally). Different to other frequently measured laboratory values such as hematological cell counts or creatinine, most patients had a distinct CRP maximum around ICU admission in our cohort, indicating a correlation with COVID-19 severity and progression (Figure 1D). Some patients showed further CRP maxima during their ICU stay, probably resulting from coinfections or secondary damage (12). We found that longitudinal data alignment based on the first local CRP maximum (CRP_max_) decreased both interpatient variability in the CRP curve (Figure 1D) and in the variability of other laboratory values such as total leukocyte and relative neutrophil and lymphocyte counts to a similar extent than the clinically based ICU admission alignment (Supplementary Figure 1).

To test whether CRP_max_-based synchronization improves patient stratification in our ICU patient cohort, we retrospectively defined three severity subgroups: (1) deceased ICU patients (*n* = 6), (2) discharged ICU patients that had been mechanically ventilated (*n* = 13) and (3) discharged ICU patients that had been spontaneously breathing while in the ICU (*n* = 9). CRP peak values were more than three-fold higher in the mechanically ventilated patient subgroups (mean ± SD, 346 ± 147 mg/L) as compared to the spontaneously breathing subgroup (99 ± 74 mg/L), but did not differ from the deceased subgroup (338 ± 106 mg/L) (Figure 1E). This lack of distinction is reflected in all alignment methods. In accordance to current literature, we further assessed the longitudinal progression of relative neutrophils and lymphocyte counts (Supplementary Figure 2) and the ratio thereof (NLR, Figure 1E) in the three severity subgroups (3, 4). While both ICU admission-based and CRP_max_-based alignment improved subgroup separation, only CRP_max_-based synchronization revealed a distinct NLR turning point, occurring simultaneously with CRP_max_, thereby providing a window for maximal subgroup distinction. A linear mixed effect model (13) employing subgroup and time as fixed effects and per-patient random slopes as random effects confirmed a difference between the subgroups and the measured time points in a window of ±4 days around CRP_max_ (*p* < 0.01, Supplementary Table 1), whereas the time wise difference was not detected in the data shifted by ICU admission (Supplementary Table 2). Similarly, when comparing the subgroups in single time points of each alignment method, only the CRP_max_-based synchronization resulted in a significant difference between the two most severe patient subgroups (Figure 1F).

**Figure 2:**
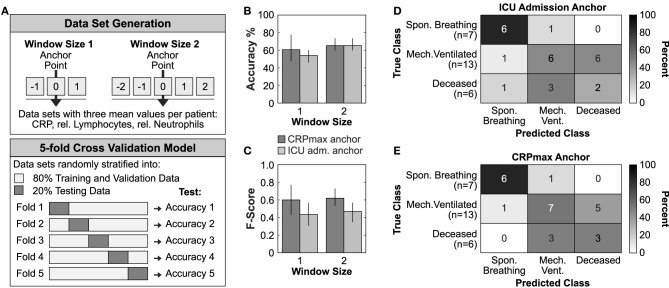
Timer-based risk stratification could improve outcome prediction. **(A)** Graphical representation of the data set generation and the applied 5-fold cross validation model. **(B,C)** Mean accuracy performance **(B)** and mean Macro-f1 score **(C)** of ICU admission or CRP_max_ anchoring. Data is reported as mean ± SD. **(D,E)** Confusion matrices constructed from the best performing trained model of each fold using the test data from all 5-folds of the ICU admission anchored **(D)** or CRP_max_ anchored **(E)** window size 1 data set.

In a last step, we explored whether different patient synchronization methods might have an impact on future outcome prediction using machine learning techniques (Figure 2). We generated two feature vectors for each patient containing the mean values of CRP, relative neutrophils and lymphocytes of a time window anchored on either ICU admission or on CRP_max_ (Figure 2A, upper panel). Using a stratified 5-fold cross validation logistic regression model, we found that the CRP_max_ anchoring increased the overall prediction accuracy by 9.6% and F1-macro score by 51.8% (accuracy 0.68 ± 0.22, F-score 0.668 ± 0.23) as compared to the ICU admission anchoring (accuracy 0.62 ± 0.10, F-score 0.44 ± 0.13) (Figures 2B,C, window size 1). Similarly, the corresponding confusion matrices indicated a higher accuracy in distinguishing between the most severe subgroups of mechanically ventilated and deceased ICU patients (Figures 2D,E).

## Discussion

We demonstrated that longitudinal data synchronization based on the inflammatory marker CRP reduces interpatient variability at least to an equal extend as the ICU admission based alignment. This pilot study is limited due to the monocentric design and the low numbers of COVID-19 patients (*n* = 28) that we were able to include during the first wave, which had a comparably mild impact on the north-eastern part of Switzerland.

Nevertheless, our study revealed that both “onset of symptoms” and “hospital admission” appear as poor temporal markers, leading to increased variability, blurring of subgroup differences and, in case of “onset of symptoms,” to patient exclusions due to unclear data. The interpretation and translation of noteworthy symptoms from patients to clinicians make “onset of symptoms” a highly subjective value for patient synchronization, which is reflected in our data and early reports of exaggerated incubation periods until onset of disease (5, 14). While ICU admission is a consistent clinical marker in our monocentric study, this might not be the case when comparing patients from different hospitals with less stringent or deviating ICU admission criteria, resources or ICU capacity. Furthermore, COVID-19 associated symptoms might not be the primary reason for ICU admission in some patients and, obviously, this temporal marker cannot be applied to non-ICU patients. These problems are encountered by most medical centers and researchers alike and highlight the necessity for an objective temporal marker that synchronizes individual patient trajectories with the underlying pathophysiology (8). Our findings suggest that in case of COVID-19, CRP can serve as such a marker that allows alignment of disease trajectories independent of hospital specific policies. We therefore encourage multicentric studies that aim at reproducing the results of this pilot study with a special focus on non-ICU ward patients. Further studies that incorporate data from subsequent COVID waves should address whether changes in patient dispositions and or treatments, such as the early administration of anti-inflammatory therapy, may limit the informative value of CRP.

In line with previous literature, our subgroup analysis of both CRP_max_ and ICU aligned data reproduced the COVID-19 severity markers: neutrophilia, lymphocytopenia and the ratio thereof (3–7). However, only CRP_max_-based longitudinal alignment improved distinction between the most severe subgroups of mechanically ventilated patients and deceased patients. Although this pilot study relies on a small cohort, our data suggests a central role for CRP in the timing of COVID-19 immunopathology by marking the turning point of longitudinal NLR dynamic and thereby providing a window for maximal subgroup distinction. CRP is under direct transcriptional control of IL-6, but shows slower dynamics, making it more likely that its maximum can be captured by daily measurements and when the patient is hospitalized (12). Interestingly, CRP itself has immune-modulating functions such as complement activation, regulation of apoptosis and cellular processes of both neutrophils and monocyte-derived cells (12). Although an elevation of CRP is generally associated with bacterial rather than viral infections (12, 15, 16), elevated CRP levels have been observed in COVID-19 patients as well as in severe progression of other respiratory viral diseases such as influenza (3, 4, 6, 17, 18). It is tempting to speculate that elevation of CRP in severe respiratory viral infections marks a shift from a more localized inflammation of the lungs to a multi-organ systemic immune response.

Digitalization of modern medicine has led to increased availability of continuous patient data that should be used to describe and define longitudinal disease progression and pathophysiology of novel and known diseases alike. Our data highlights that proper synchronization of longitudinal patient data has the potential to improve mortality-risk stratification and subgroup distinction both in a clinical setting and for research purposes.

## Data Availability Statement

The data analyzed in this study is subject to the following licenses/restrictions: data protection laws. Requests to access these datasets should be directed to jan.bartussek@usz.ch.

## Ethics Statement

The studies involving human participants were reviewed and approved by Cantonal Ethics Committee Zurich. The Ethics Committee waived the requirement of written informed consent for participation.

## Author Contributions

MM and JB conceptualized and performed the research and wrote the manuscript. AA performed the 5-fold cross-validation modeling. MH performed the mixed linear regression modeling and RISC-19-ICU registry data analysis. The CoViD-19 ICU-Research Group Zurich and the RISC-19-ICU Investigators contributed to the data collection. AA, SB, PB, CG, NP, MH, MK, RS, and PW provided valuable input and critically assessed the manuscript. All authors contributed to the article and approved the submitted version.

## The COVID-19 ICU-Research Group Zurich

University Hospital of Zurich, Institute for Intensive Care: Bartussek, Jan; Buehler, Phillip; Heuberger, Dorothea Monika; Hilty, Matthias Peter; Hofmänner, Daniel Andrea; Maibach, Martina Anna; Schuepbach Reto Andreas; Wendel Garcia, Pedro David. University Hospital of Zurich, Department of Infectious Diseases and Hospital Epidemiology: Brugger, Silvio; Mairpady Shambat, Srikanth; Zinkernagel, Annelies.

## THe RISC-19-ICU Investigators

**Andorra:** Unidad de Cuidados Intensivos, Hospital Nostra Senyora de Meritxell, Escaldes-Engordany (Mario Alfaro-Farias; Gerardo Vizmanos-Lamotte). **Austria:** Department of Anesthesiology and Critical Care Medicine, Kepler University Hospital GmbH and Johannes Kepler University, Linz (Thomas Tschoellitsch; Jens Meier). **Ecuador:** Unidad de Cuidados Intensivos, Hospital Vicente Corral Moscoso, Cuenca (Hernán Aguirre-Bermeo; Janina Apolo; Alberto Martínez). **France:** SCPARE-Intensive Care Unit, Clinique Louis Pasteur, Essey-lès-Nancy (Geoffrey Jurkolow; Gauthier Delahaye); Department of Anesthesiology and Critical Care Medicine, University Hospital of Nancy, Nancy (Philippe Guerci; Emmanuel Novy; Marie-Reine Losser). **Germany:** Department of Medicine III - Interdisciplinary Medical Intensive Care, Medical Center University of Freiburg, Freiburg (Tobias Wengenmayer; Jonathan Rilinger; Dawid L. Staudacher); Medical Intensive Care, Medical School Hannover, Hannover (Sascha David; Tobias Welte; Klaus Stahl). **Greece:** Intensive Care Unit, St. Paul (“Agios Pavlos”) General Hospital of Thessaloniki, Thessaloniki (Theodoros Aslanidis). **Hungary:** Department of Anesthesiology and Intensive Care, University of Szeged (Anita Korsos; Barna Babik). **Iran:** Intensive Care Department, Shiraz University of Medical Sciences, Shiraz (Reza Nikandish). **Italy:** Department of Anesthesia and Intensive Care Medicine, Policlinico San Marco, Gruppo Ospedaliero San Donato, Bergamo (Emanuele Rezoagli; Matteo Giacomini; Alice Nova); Anesthesia and Intensive care, Azienda Ospedaliero-Universitaria di Ferrara, Cona (Alberto Fogagnolo; Savino Spadaro); UO Anestesia e Terapia Intensiva, IRCCS Centro Cardiologico Monzino, Milan (Roberto Ceriani; Martina Murrone); Department of Internal Medicine, ASST Fatebenefratelli Sacco - “Luigi Sacco” Hospital, Milan (Maddalena A. Wu; Chiara Cogliati); Division of Anesthesia and Intensive Care, ASST Fatebenefratelli Sacco - “Luigi Sacco” Hospital, Milan (Riccardo Colombo; Emanuele Catena); Internal Medicine, Azienda Ospedaliera Universitaria di Modena, Modena (Fabrizio Turrini; Maria Sole Simonini; Silvia Fabbri); UOC Anestesia e Rianimazione, Ospedale Infermi, Rimini (Jonathan Montomoli; Antonella Potalivo; Francesca Facondini); U.O Pronto Soccorso - Medicina d'Urgenza, Ospedale Infermi, Rimini (Gianfilippo Gangitano; Tiziana Perin); Department of Anaesthesia and Intensive Care, Fondazione Policlinico Universitario A. Gemelli IRCCS, Università Cattolica del Sacro Cuore, Rome, Italy (Maria Grazia Bocci; Massimo Antonelli). **Netherlands:** Department of Intensive Care Medicine, Erasmus Medical Center, Rotterdam (Diederik Gommers; Can Ince). **Spain:** Servicio de Medicina intensiva, Complejo Hospitalario Universitario A Coruña, A Coruña (Raquel Rodríguez-García; Jorge Gámez-Zapata; Xiana Taboada-Fraga); Medical Intensive Care Unit, Hospital Clínic de Barcelona, Barcelona (Pedro Castro; Adrian Tellez); Servicio de Medicina Intensiva, Hospital General San Jorge, Huesca (Arantxa Lander-Azcona; Jesús Escós-Orta); Servicio de Medicina Intensiva, Hospital Universitario de Torrejon, Madrid (Maria C. Martín-Delgado; Angela Algaba-Calderon); Servicio de Medicina intensiva, Hospital Verge de la Cinta, Tortosa (Diego Franch-Llasat; Ferran Roche-Campo); Unidad de Cuidados Intensivos, Hospital Clínico Universitario Lozano Blesa, Zaragoza (Herminia Lozano-Gómez; Begoña Zalba-Etayo). **Switzerland:** Medizinische Intensivstation, Kantonsspital Aarau, Aarau (Marc P. Michot, Alexander Klarer); Klinik für Operative Intensivmedizin, Kantonsspital Aarau, Aarau, Switzerland (Rolf Ensner); Institut fuer Anesthaesie und Intensivmedizin, Zuger Kantonsspital AG, Baar (Peter Schott; Severin Urech); Department Intensivmedizin, Universitaetsspital Basel, Basel (Nuria Zellweger); Intensivmedizin, St. Claraspital, Basel (Lukas Merki, MD; Adriana Lambert); Department Intensive Care Medicine, Spitalzentrum Biel, Biel, Switzerland (Marcus Laube); Department of Intensive Care Medicine, University Hospital Bern, Inselspital, Bern (Marie M. Jeitziner; Beatrice Jenni-Moser); Interdisziplinaere Intensivmedizin, Lindenhofspital, Bern, Switzerland (Jan Wiegand); Interdisziplinaere Intensivstation, Spital Buelach, Buelach (Bernd Yuen; Barbara Lienhardt-Nobbe; AndreaWestphalen); Intensivstation, Regionalspital Emmental AG, Burgdorf (Petra Salomon; Iris Drvaric); Intensivmedizin, Kantonsspital Graubuenden, Chur (Frank Hillgaertner; Marianne Sieber); Institut fuer Anaesthesie und Intensivmedizin, Spital Thurgau, Frauenfeld (Alexander Dullenkopf; Lina Petersen; Ivan Chau); Soins Intensifs, Hopital cantonal de Fribourg, Fribourg (Hatem Ksouri; Govind Oliver Sridharan); Division of Intensive Care, University Hospitals of Geneva, Geneva (Sara Cereghetti; Filippo Boroli; Jerome Pugin); Division of Neonatal and Pediatric Intensive Care, University Hospitals of Geneva, Geneva (Serge Grazioli; Peter C. Rimensberger); Intensivstation, Spital Grabs, Grabs, Switzerland (Christian Bürkle); Institut für Anaesthesiologie Intensivmedizin and Rettungsmedizin, SeeSpital Horgen and Kilchberg, Horgen (Julien Marrel; Mirko Brenni); Soins Intensifs, Hirslanden Clinique Cecil, Lausanne (Isabelle Fleisch; Jerome Lavanchy); Pediatric Intensive Care Unit, University Hospital Lausanne, Lausanne (Marie-Helene Perez; Anne-Sylvie Ramelet); Anaesthesie und Intensivmedizin, Kantonsspital Baselland, Liestal (Anja Baltussen Weber; Peter Gerecke; Andreas Christ); Dipartimento Area Critica, Clinica Luganese Moncucco, Lugano (Samuele Ceruti; Andrea Glotta); Interdisziplinaere Intensivstation, Spital Maennedorf AG, Maennedorf (Katharina Marquardt; Karim Shaikh); Institut fuer Anaesthesie und Intensivmedizin, Spital Thurgau, Muensterlingen (Tobias Hübner; Thomas Neff); Intensivmedizin, Schweizer Paraplegikerzentrum Nottwil, Nottwil, Switzerland (Hermann Redecker); Soins intensifs, Groupement Hospitalier de l'Ouest Lémanique, Hôpital de Nyon, Nyon (Thierry Fumeaux; Mallory Moret-Bochatay); Klinik für Anaesthesie und Intensivmedizin, Spitalzentrum Oberwallis, Visp, Switzerland (Friederike Meyer zu Bentrup) Intensivmedizin and Intermediate Care, Kantonsspital Olten, Olten, Switzerland (Michael Studhalter); Intensivmedizin, Spital Oberengadin, Samedan (Michael Stephan; Jan Brem); Intensivstation, Kantonsspital Schaffhausen, Schaffhausen, Switzerland (Nadine Gehring); Anaesthesie Intensivmedizin Schmerzmedizin, Spital Schwyz, Schwyz (Daniela Selz; Didier Naon); Medizinische Intensivstation, Kantonsspital St. Gallen, St. Gallen, Switzerland (Gian-Reto Kleger); Departement of Anesthesiology and Intensive Care Medicine, Kantonsspital St. Gallen, St. Gallen (Urs Pietsch; Miodrag Filipovic); Department for Intensive Care Medicine, Kantonsspital Nidwalden, Stans (Anette Ristic; Michael Sepulcri); Intensivstation, Spital Simmental-Thun-Saanenland AG, Thun, Switzerland (Antje Heise); Service d'Anesthesiologie, EHNV, Yverdonles-Bains (Marilene Franchitti Laurent; Jean-Christophe Laurent); Institute of Intensive Care Medicine, University Hospital Zurich, Zurich (Pedro D. Wendel Garcia; Matthias Hilty; Reto Schuepbach; Dorothea Heuberger; Philipp Bühler; Silvio Brugger); Interdisziplinaere Intensivstation, Stadtspital Triemli, Zurich (Patricia Fodor; Pascal Locher; Giovanni Camen); Abteilung für Anaesthesiologie und Intensivmedizin, Hirslanden Klinik Im Park, Zürich (Tomislav Gaspert; Marija Jovic); Institut für Anaesthesiologie und Intensivmedizin, Klinik Hirslanden, Zurich (Christoph Haberthuer; Roger F. Lussman). **Turkey:** General Surgery, Samsun Training and Research Hospital, Samsun (Elif Colak).

## Conflict of Interest

The authors declare that the research was conducted in the absence of any commercial or financial relationships that could be construed as a potential conflict of interest.

## Supplementary Material

Supplementary Figure 1 Dynamic changes of CRP, leukocytes and relative neutrophils and lymphocytes. Longitudinal CRP, leukocyte, neutrophils and lymphocytes shifted relative to symptom onset (left), hospital admission (middle, left), ICU admission (middle, right) or first local CRP maximum (right). Synchronization based on onset of symptoms resulted in the exclusion of two deceased patients due to unclear data. Data is shown as median ± MAD. Curves are cut-off when data of fewer than three patients was available. The respective patient numbers are shown in the bottom panels.

Supplementary Figure 2 Dynamic changes of leukocytes, relative and absolute lymphocytes and neutrophils of severity subgroups. Patients were retrospectively divided into three severity subgroups: spontaneously breathing (blue, *n* = 9), mechanically ventilated (yellow, *n* = 13) and deceased patients (red, *n* = 6). Longitudinal data is synchronized based on onset of symptoms (left) hospital admission (middle, left), ICU admission (middle, right) or first local CRP maximum (right). Synchronization based on onset of symptoms resulted in the exclusion of two deceased patients due to unclear data. Data is shown as median ± MAD. Curves are cut-off when data of fewer than three patients was available. The respective patient numbers are shown in the bottom panels.

Supplementary Table 1 Mixed linear regression model for ICU shifted data. *P*-Value indicate likelihood ratio test for overall analysis and Satterthwaite approximation for subgroup analysis.

Supplementary Table 2 Mixed linear regression model for CRPmax shifted data. *P*-Value indicate likelihood ratio test for overall analysis and Satterthwaite approximation for subgroup analysis.
