# Supplementary material for: How to Synchronize Longitudinal Patient Data With the Underlying Disease Progression: A Pilot Study Using the Biomarker CRP for Timing COVID-19
Source: Front Med (Lausanne). 2021 Jul 8;8:607594. doi: 10.3389/fmed.2021.607594 (PMC8295502; doi:10.3389/fmed.2021.607594)
Supplement: Supplementary Figure 1 — Dynamic changes of CRP, leukocytes and relative neutrophils and lymphocytes. Longitudinal CRP, leukocyte, neutrophils and lymphocytes shifted relative to symptom onset (left), hospital admission (middle, left), ICU admission (middle, right) or first local CRP maximum (right). Synchronization based on onset of symptoms resulted in the exclusion of two deceased patients due to unclear data. Data is shown as median ± MAD. Curves are cut-off when data of fewer than three patients was available. The respective patient numbers are shown in the bottom panels. [file Data_Sheet_1.pdf]

Supplementary Material: How to synchronize longitudinal patient data with the underlying disease progression: A pilot study using the biomarker CRP for timing COVID-19

Martina A. Maibach[1], Ahmed Allam[2], Matthias P. Hilty[1], Nicolas A. Perez Gonzalez[2], Philipp K. Buehler[1], Pedro D. Wendel Garcia[1], Silvio D. Brugger[3], Christoph C. Ganter[1], The CoViD-19 ICU-Research Group Zurich, The RISC-19-ICU Investigators, Michael Krauthammer[2,*], Reto A. Schuepbach[1,*] and Jan Bartussek[1,2,*]

[1] *Institute for Intensive Care Medicine, University and University Hospital Zurich, Switzerland*
[2] *Department of Quantitative Biomedicine, University and University Hospital Zurich, Switzerland*
[3] *Department of Infectious Diseases and Hospital Epidemiology, University and University Hospital Zurich, Switzerland*

[*] *michael.krauthammer@usz.ch, reto.schuepbach@usz.ch, jan.bartussek@usz.ch*

Content

This document contains:

1. Supplementary figure 1: *Dynamics of CRP, leukocytes, neutrophils and lymphocytes*
2. Supplementary figure 2: *Dynamics of CRP, leukocytes, neutrophils and lymphocytes*
3. Supplementary table 1: *Mixed linear regression model for* CRP_{max} *shifted data*
4. Supplementary table 2: *Mixed linear regression model for ICU shifted data*

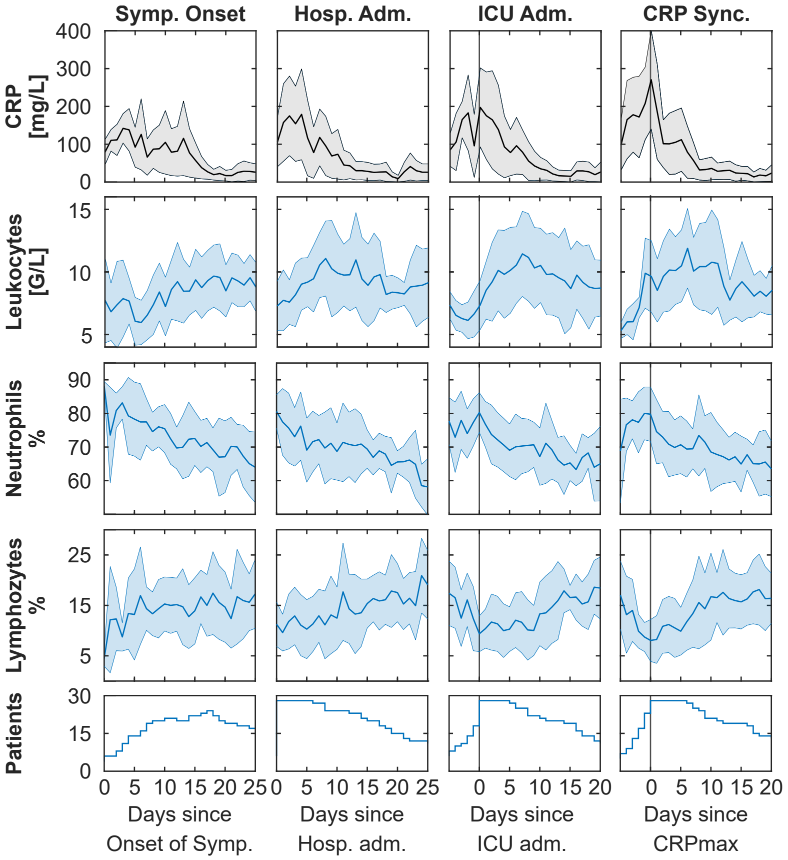

Supplementary Figure 1: Dynamics CRP, leukocytes and relative neu-trophils and lymphocytes. Longitudinal CRP, leukocyte, neutrophils and lympho-cytes shifted relative to symptom onset (left), hospital admission (middle, left), ICU admission (middle, right) or first local CRP maximum (right). Synchronization based on onset of symptoms resulted in the exclusion of two deceased patients due to unclear data. Data is shown as median ± MAD. Curves are cut-off when data of fewer than three patients was available. The respective patient numbers are shown in the bottom panels.

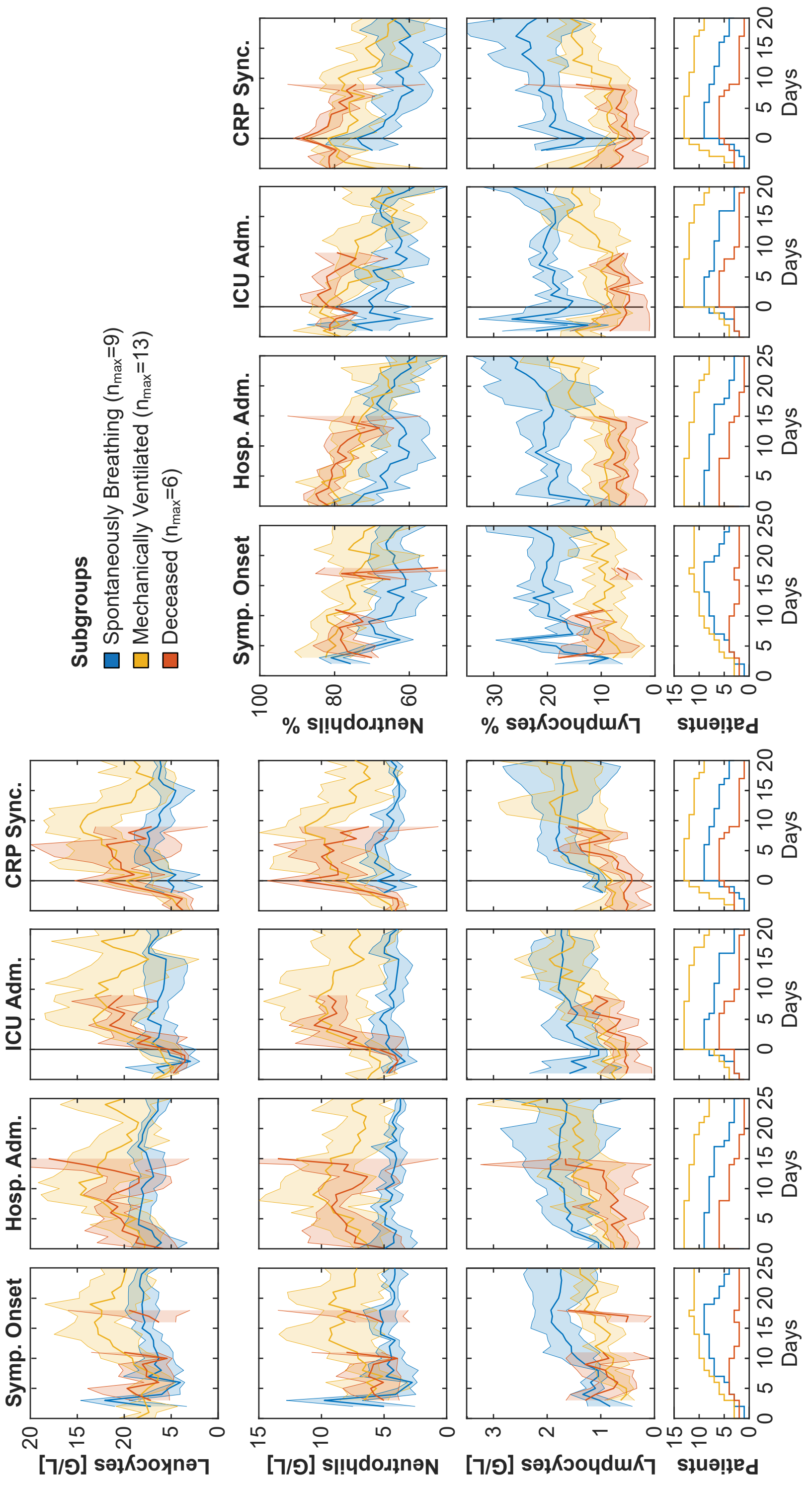

Supplementary Figure 2: Subgroup specific dynamics of leukocytes, rela-tive and absolute lymphocytes and neutrophils. Patients were retrospectively divided into three severity subgroups: spontaneously breathing (blue, n=9), mechanically ventilated (yellow, n=13) and deceased patients (red, n=6). Longitudinal data is synchronized based on onset of symptoms (left) hospital admission (middle, left), ICU admission (middle, right) or first local CRP maximum (right). Synchronization based on onset of symptoms resulted in the exclusion of two deceased patients due to unclear data. Data is shown as median ± MAD. Curves are cut-off when data of fewer than three patients was available. The respective patient numbers are shown in the bottom panels.

NLR μ ± SD,	Spont. Breathing (n=9)	Mech. Vent (n=13)	Deceased (n=6)	Time estimates ± S.E. (CI)	Degrees of freedom	p time
day -4	-	9.87 ± 8.84	28.19 ± 32.41			
day -2	6.34 ± 4.58	10.33 ± 5.88	30.06 ± 27.67	22.32 ± 6.04 (12.15 - 32.49)	92.36	0.222
day 0	4.79 ± 2.36	16.46 ± 19.83	38.22 ± 37.1	7.7 ± 3.3 (2.21 - 13.16)	92.65	**0.022**
day 2	3.87 ± 1.16	10.22 ± 6.99	23.54 ± 22.36	1.36 ± 3.3 (-4.12 - 6.83)	92.65	0.681
day 4	3.92 ± 1.67	10.23 ± 5.11	14.21 ± 13.3	-0.62 ± 3.3 (-6.1 - 4.85)	92.65	0.852
Group estimates ± S.E. (CI)		7.2 ± 4.98 (-1.18 - 15.59)	22.32 ± 6.04 (12.15 - 32.49)			**< 0.01**
Degrees of freedom		29.48	29.19			
p group		0.159	**< 0.001**	< 0.01		

Supplementary Table 1: Mixed linear regression model for CRP$_{max}$ shifted data. P-Value indicate likelihood ratio test for overall analysis and Satterthwaite approximation for subgroup analysis.

NLR μ ± SD,	Spont. Breathing (n=9)	Mech. Vent (n=13)	Deceased (n=6)	Time estimates ± S.E. (CI)	Degrees of freedom	p time
day -4	5.99 ± 4.1	8.13 ± 4.72	33.01 ± 42.28			
day -2	5.26 ± 3.79	12.77 ± 5.91	30.16 ± 36.4	-0.97 ± 3.65 (-7.01 - 5.08)	85.10	0.791
day 0	4.41 ± 1.97	14.36 ± 14.66	30.69 ± 33.53	1.19 ± 3.48 (-4.59 - 6.96)	86.85	0.734
day 2	3.57 ± 0.96	13.19 ± 12.52	17.08 ± 12.81	-2.55 ± 3.48 (-8.33 - 3.22)	86.85	0.466
day 4	4.01 ± 1.65	11.62 ± 11.83	24.03 ± 18.56	-1.75 ± 3.52 (-7.58 - 4.08)	87.17	0.620
Group estimates ± S.E. (CI)		7.87 ± 4.96 (-0.5 - 16.21)	20.93 ± 6.02 (10.77 - 31.07)			0.600
Degrees of freedom		28.46	28.44			
p group		0.124	**0.002**	**< 0.01**		

Supplementary Table 2: Mixed linear regression model for ICU shifted data. P-Value indicate likelihood ratio test for overall analysis and Satterthwaite approximation for subgroup analysis.
